# *Satureja bachtiarica* Induces Cancer Cell Death in Breast and Glioblastoma Cancer in 2D/3D Models and Suppresses Breast Cancer Stem Cells

**DOI:** 10.3390/cells12232713

**Published:** 2023-11-26

**Authors:** Vajihe Azimian Zavareh, Shima Gharibi, Mahnaz Hosseini Rizi, Abdolhossein Nekookar, Hossein Mirhendi, Mehdi Rahimmalek, Antoni Szumny

**Affiliations:** 1 Core Research Facilities (CRF), Isfahan University of Medical Sciences, Isfahan 81746-73461, Iran; v.azimian@mail.mui.ac.ir (V.A.Z.); s.gharibi@mail.mui.ac.ir (S.G.); mahnaz.h1392@gmail.com (M.H.R.); nekookar.vet@gmail.com (A.N.); s.h.mirhendi@gmail.com (H.M.); 2 Department of Pharmaceutical Biology and Biotechnology, Wroclaw Medical University, 50-367 Wrocław, Poland; 3 Department of Medical Parasitology and Mycology, School of Medicine, Isfahan University of Medical Sciences, Isfahan 81746-73461, Iran; 4 Department of Horticulture, College of Agriculture, Isfahan University of Technology, Isfahan 84156-83111, Iran; 5 Department of Food Chemistry and Biocatalysis, Wroclaw University of Environmental and Life Sciences, 50-375 Wrocław, Poland

**Keywords:** cancer stem cells, *Satureja bachtiarica*, medicinal plants, spheroid, breast cancer, glioblastoma

## Abstract

Overcoming drug resistance and specifically targeting cancer stem cells (CSCs) are critical challenges in improving cancer therapy. Nowadays, the use of novel and native medicinal plants can provide new sources for further investigations for this purpose. The aim of this study was to assess the potential of *S. bachtiarica*, an endemic plant with diverse medicinal applications, in suppressing and targeting cancer and cancer stem cells in glioblastoma and breast cancer. The effect of *S. bachtiarica* on viability, migration, invasion, and clonogenic potential of MDAMB-231 and U87-MG cells was assessed in both two- and three-dimensional cell culture models. Additionally, we evaluated its effects on the self-renewal capacity of mammospheres. The experimental outcomes indicated that *S. bachtiarica* decreased the viability and growth rate of cells and spheroids by inducing apoptosis and inhibited colony formation, migration, and invasion of cells and spheroids. Additionally, colony and sphere-forming ability, as well as the expression of genes associated with EMT and stemness were reduced in mammospheres treated with *S. bachtiarica*. In conclusion, this study provided valuable insights into the anti-cancer effects of *S. bachtiarica*, particularly in relation to breast CSCs. Therefore, *S. bachtiarica* may be a potential adjuvant for the treatment of cancer.

## 1. Introduction

Cancer, a highly invasive and destructive disease, ranks as the second leading cause of death globally following cardiovascular diseases [1], particularly affecting developing countries with lower survival rates due to delayed diagnoses and limited treatment access. Current cancer treatments, including chemotherapy, radiation, and surgery, often encounter limitations such as severe side effects and harm to healthy tissues [2]. The primary treatment goal is complete cancer removal while minimizing damage to normal tissues. One of the significant hurdles in cancer therapy is drug resistance, which adversely affects patient survival rates and the effectiveness of anti-cancer drugs [3]. This resistance is multifaceted, involving factors like genetic mutations that promote various cancer cell populations and the existence of cancer stem cells (CSCs) [4,5]. CSCs, a subset of tumor cells with self-renewal abilities, play a critical role in tumor initiation and growth maintenance. Accumulating evidence has proved that CSCs are the driving force behind breast cancer [6] and glioblastoma (GBM) [7] tumor formation, progression, metastasis, therapeutic resistance, and recurrence. Therefore, targeting and eradicating these cells could potentially reduce the risk of relapse and contribute to better treatment outcomes [8,9,10].

Given the limitations of conventional methods, such as the inability of two-dimensional (2D) cell cultures to replicate the complex tumor environment and the cost and time demands of animal xenograft models, the focus has shifted towards three-dimensional (3D) cell cultures, particularly sphere culture to enhance CSCs [10]. Therefore, in this study, we utilized both 2D and 3D cell culture models, as well as sphere culture, to investigate cancer and CSCs’ behavior.

Considering the role of CSCs in treatment resistance, the development of compounds that specifically target CSCs holds promise as more effective therapeutic agents for cancer treatment, aiming to control recurrence and prevent metastasis.

In recent years, there has been a surge of interest among scientists in natural metabolites derived from medicinal plants. These metabolites have garnered attention for their safety profile, ability to target both diverse cancer cell populations and CSCs, and their modulation of essential signaling pathways [11]. Reports indicate that plant nutraceuticals can combat breast cancer by reducing breast cancer severity, restricting malignant cell growth, and modifying cancer-related mechanisms such as the attenuation of Hedgehog, Nuclear factor kappa-light-chain-enhancer of activated B cells (NF-κB), Notch, and Wnt/β-catenin signaling as key signaling pathways involved in breast CSCs self-renewal [12,13]. In aggressive brain cancers, medicinal herbs can also trigger tumor cells, regulate their anti-cancer mechanisms and immune responses to assist in cancer elimination, and cause cell death [14]. Given the rich diversity of medicinal plants indigenous to Iran, they represent a promising avenue for exploring new compounds and products that could effectively target CSCs and contribute to advancements in cancer treatment.

The genus *Satureja* comprises approximately 200 species worldwide. In the Iranian flora, sixteen species have been reported, nine of them are native to Iran [15]. Among these species, *S. bachtiarica* is considered as an endemic plant with various applications in food and pharmacy [16]. In Iranian traditional medicine, *S. bachtiarica* is utilized as a carminative, appetizer, and sexual enhancer. It is also recommended for the treatment of conditions such as cough, dyspnea, diarrhea, and stomach cramps [17]. Pharmacological studies have demonstrated several beneficial properties of the *S. bachtiarica* extract. These include antioxidant and radical scavenging effects [18], potential anticancer activity [19], and neuroprotective properties [20], as well as anti-inflammatory and analgesic effects [21]. Moreover, this species is introduced as an immunostimulant without causing hematological side effects [22]. Finally, anti-*Helicobacter pylori* effect of *S. bachtiarica* was also reported [23].

In addition to some pharmaceutical research on *S. bachtiarica*, there is no documented study with regards to the anti-breast cancer and glioblastoma properties of this valuable species. In the present study, glioblastoma and breast cancer cell lines were utilized in both 2D and 3D models, including multicellular spheroids and spheres. The aim was to investigate the impact of *S. bachtiarica* on various aspects of cancer cell behavior, such as viability, migration, invasion, and colony formation potential in both models. Additionally, mammospheres were employed as a model for breast cancer stem cells to evaluate the impact of *S. bachtiarica* on their self-renewal capacity. The findings of this research contribute to a better understanding of the mechanisms underlying the anti-cancer effects of *S. bachtiarica* in glioblastoma and breast cancer. Moreover, it has the potential to enhance our ability to specifically target cancer stem cells, which perform a crucial role in tumor development and progression. Finally, the new potential endemic plant can be introduced for such anti-cancer effects.

## 2. Materials and Methods

### 2.1. Plant Materials

The *S. bachtiarica* aerial parts were collected in Naghan, Chahar Mahal-e Bakhtiari (31°98′ N and 50°68′ N) at an altitude of 2026 meters with herbarium number of 13,344. The samples were collected and identified by Dr. Mehdi Rahimmalek using Flora Iranica (Rechinger, 1963), and the samples were deposited in the herbarium of Isfahan University of Technology, Isfahan, Iran. The collected plants were dried at 25 °C for three days in shade condition.

### 2.2. HPLC Analysis

*S. bachtiarica* aerial parts were used for polyphenolic compound determination based on standards including gallic acid, vanillic acid, caffeic acid, ferulic acid, *p*-coumaric acid, quercetin, luteolin, and apigenin (Phytolab, Germany, 98% purity). Methanolic extraction was applied, in which 2g of leaf samples was used and after grinding were extracted using 20 mL of methanol (80%), and shaken at 25 °C for 20 h. The extraction was repeated twice and the air-dried extract was dissolved in HPLC solvent A (1 mL), filtered (0.22 μm disk), and 20 μL was injected to an Agilent 1090 system with detection range of 260 and 350 nm. A 250 × 4.6 mm, 5 μm, symmetry C18 column (Waters Crop., Milford, MA, USA) was applied in this experiment. The mobile phase included formic acid (99.9:0.1) as a solution (A) and acetonitrile/formic acid (99.9:0.1) as a solution (B) with gradient elution at 25 °C and flow rate of 0.8 mL min^−1^. The gradient program started from A: B (90:10) for 1 min, followed by 10–26% B for 40 min, 26–65% B for 30 min, and finally 65–100% B for 5 min followed by equilibration with 0–90% A for 4 min. Polyphenolic components were determined by comparing UV spectra and the retention times with pure standards, and the amount was reported in mg per 100 g of dry sample weight.

### 2.3. Cell lines and Culture Conditions

In this study, three cell lines were used: MDAMB-231 as a poorly differentiated triple-negative breast cancer (TNBC) cell line; U87-MG as a human glioblastoma cell line; and human lung fibroblast cell line (MRC-5) as a normal cell line. All the human cell lines were obtained from the Pasteur Institute of Iran. U87-MG was cultured in a high glucose Dulbecco’s modified Eagle’s medium (DMEM; Gibco BRL, Paisley, UK) and MDAMB-231 and MRC-5 cell lines were cultivated in DMEM-F12 (Gibco BRL, Paisley, UK) supplemented with 10% fetal bovine serum (FBS), 100 U/mL penicillin, and 100 µg/mL streptomycin (all obtained from Life Technologies GmbH, Darmstadt, Germany). Cells were incubated in a humidified 5% CO_2_ incubator at 37 °C. Trypsin/EDTA (Invitrogen; Thermo Fisher Scientific, Inc., Santa Barbara, CA, USA.) was used to harvest the cells.

### 2.4. Spheroid Culture

Uniformity in spheroid size and number of cells incorporated into a spheroid for effective drug treatment is important. Due to this, we used the hang drop method and drops of 50 µL containing 2000 cells suspended in cell culture media with 10% FBS, 1% NEAA, 1% L-glutamine, and 1% penicillin/streptomycin and placed drops on the inner side of the lid of 100 mm tissue culture dishes. Then, dishes were incubated under standard conditions (5% CO_2_, 37 °C). After two days, MDAMB-231 and U87-MG spheroids were transferred into agarose-coated 24-well plates. The media were changed every two days.

### 2.5. Sphere Culture

We used an anchorage-independent method for sphere (glioblastoma and mammosphere) culture. T25 flasks were coated with 1% agarose and washed once with PBS before cell seeding. A total of 10 × 10^4^ viable cells were cultured in non-adherent T25 flasks in serum-free medium supplemented with 20 ng/mL of epithelial growth factor (EGF) and basic fibroblast growth factor (bFGF) obtained from the Royan Institute in Tehran, Iran, as well as 2% B27 from Thermo Fisher Scientific, Inc. EGF, bFGF, and B27 were added every other day.

### 2.6. Cell Viability Assay

We used 3-(4,5-dimethylthiazol-2-yl)-2,5-diphenyltetrazolium bromide (MTT) dye (Sigma-Aldrich GmbH; Saint Louis, USA) for cell proliferation assay. All cell lines were seeded at a density of 1 × 10^4^ cells/well in 96-well plates with 100 μL medium in each well. After culturing overnight, the dried *S. bachtiarica* extract was dissolved in DMSO and various concentrations of *S. bachtiarica* (0 as control, 100, 200, 500, 800, and 1000 μg/mL) were prepared, and then cells were treated with them for 24 and 48 h. At the end of incubation times, MTT stock solution (5 mg/mL) was added to the incubated cells (10 μL/well), the cells were then incubated for 4 h at 37 °C, and subsequently MTT-formazan product was dissolved by adding 100 µL dimethyl sulfoxide (DMSO) to each well, and the optical density of the culture medium was measured in a micro-plate reader (Bio-Tek Instruments, Ltd, Winooski, VT, USA) at 570 nm. The percentage of cell viability was calculated as follows: (Average absorbance of the treated group/average absorbance of the control group) × 100. Viability assays were performed in triplicate.

### 2.7. Spheroid Growth Rate Assay

After hanging drop, 4-day spheroids were treated with an IC_50_ dose of *S. bachtiarica* (630 μg/mL and 680 μg/mL for MDA-MB-231 and U87-MG cells, respectively). The treatment time of the spheroids was considered as day 0, and morphology of the spheroids was checked and photographed using an inverted light microscope during nine days after the treatment. The images were processed and the diameter of the individual spheroids was calculated using Image J software (1.52v). Spheroids’ media were changed every three days.

### 2.8. Annexin V/PI Double Staining Assay for Apoptosis

Apoptosis induction was assessed using Annexin V-FITC/PI apoptosis detection kit (BD Biosciences, San Jose, CA, USA), according to the manufacturer’s instructions. MDAMB-231 and U87-MG cancer cell lines were briefly seeded at a concentration of 5 × 10^5^ cells/mL in a six-well plate and incubated overnight. The cells were then treated with IC_50_ dose of *S. bachtiarica* (630 μg/mL and 680 μg/mL for MDA-MB-231 and U87-MG cells, respectively) for 48 h. After the treatment, cells were harvested, washed, and collected using centrifugation at 1500 RPM for 5 min. Cells were stained with annexin V (2 µL) and PI (2 µL) at room temperature for 10 min, and protected from light. Stained cells were analyzed using a fluorescence-activated cell sorter flow cytometer (FC 500 Series Flow Cytometry, Beckman Coulter, Indianapolis, IN, USA). A minimum of 10,000 cells were analyzed for each sample, and the experiments were conducted in triplicate.

### 2.9. Scratch Wound-Healing and Transwell Invasion Assays

For Scratch assay, MDAMB-231 and U87-MG cells with 90% confluency were serum-starved overnight, then they were treated with 10 µg/mL mitomycin for two hours (Sigma-Aldrich Chemie Gmbh, Munich, Germany). Scratches were made on the cell monolayer using a yellow tip and captured at the selected positions at intervals of 0, 3, 6, 18, and 24 h with or without low and IC_50_ doses of *S. bachtiarica* (630 μg/mL and 680 μg/mL for MDA-MB-231 and U87-MG cells, respectively). The migration rate of cells at each time point was analyzed relative to time 0. For invasion assay, 8.0 µm pore Transwell inserts (Corning Inc. Life Sciences, Tewksbury, MA, USA) was coated with 60 μL of diluted Matrigel (0.5 mg/mL; BD Biosciences, San Jose, CA, USA) for 24 h. MDAMB-231 and U87-MG cells were serum starved for overnight, and then 200 µL serum-free culture medium containing 2.5 × 10^4^ cells treated with IC_50_ dose of *S. bachtiarica* for 48 h and control cells was added to the upper chamber of filter inserts. Each filter was placed in the lower chamber with 600 µL culture medium containing 10% FBS. Filters were incubated at 37 °C with 5% CO_2_ for 12 h. The remaining cells inside the chamber were removed by gently swabbing with a cotton swab, and the cells attached to the bottom surface of filter were fixed with 4% paraformaldehyde at 4 °C for 45 min. Afterwards, these cells were stained using a 0.5% crystal violet solution for 5 min at room temperature. To determine the number of cells attached to the bottom surface of the filter, 10 random fields were selected and observed under an inverted light microscope at a magnification of 20×. The counts obtained were then adjusted relative to the control group.

### 2.10. Spheroid Disaggregation Assay

Ninety-six-well plates were coated with Matrigel overnight at 37 °C. The wells were then blocked with 2 mg/mL BSA in PBS for 1 h, then rinsed twice with PBS. Spheroids (5/well) in the presence or absence of IC_50_ dose of *S. bachtiarica* (630 μg/mL and 680 μg/mL for MDA-MB-231 and U87-MG cells, respectively) were added to each coated well for 48 h. The spheroids attached to the surface of the coated plate after 1 h, which was considered as the starting time point (t = 0), and then digital photographs of the spheroids were taken at t = 0, t = 18, t = 24, and t = 48 h. Disaggregated spheroids formed a single layer of cells, derived from the peripheral layer of spheroids. The pixel area of the disaggregated spheroids was determined at indicated time points using ImageJ software by outlining the entire area of the spheroids or the dispersed cells. The calculated total area included the area of the disaggregated spheroid, as well as the area of any dispersed single cells in the near vicinity that were most likely to have come from the disaggregated spheroid. The fold change in the area was determined by dividing the pixel area of the spheroid at 18, 24, and 48 h by the pixel area at time 0.

### 2.11. Colony Assay

For colony assay, MDAMB-231 and U87-MG cell lines, as well as mammospheres were treated with an IC_50_ dose of *S. bachtiarica* (630 μg/mL and 680 μg/mL for MDA-MB-231 and U87-MG cells, respectively) for 48 h. After that, untreated and treated cells and mammospheres were collected using trypsinization and washed with phosphate-buffered saline (PBS), counted, and plated in 6-well plates at 300 cells/well and incubated at 37 °C for 11 days. The colonies were washed with calcium and magnesium-free PBS, fixed with 4% paraformaldehyde at 4 °C for 45 min, then stained with 200 µL 0.05% crystal violet for 5 min at room temperature (RT). The colonies were counted using an inverted microscope at 10× magnification. The data are presented as the number of colonies formed with and without treatment. Clonogenicity was calculated as follows: (Average number of colonies formed/number of cells seeded) × 100.

### 2.12. Sphere Formation Efficiency Assay

For sphere formation ability assay, MDAMB-231 and U87-MG cell lines were treated with an IC_50_ dose of *S. bachtiarica* (630 μg/mL and 680 μg/mL for MDA-MB-231 and U87-MG cells, respectively) for 48 h, then cells were washed and trypsinized. A total of 2 × 10^4^ viable cells/well were cultured in 6-well plates coated with 1% agarose containing 2 mL serum free medium supplemented with EGF, bFGF, and B27. After six days, the mean diameter of the spheres was quantified with ImageJ software and the spheres with >100 μm diameter were taken for further counting using an inverted microscope at 40× magnification. Sphere formation efficiency was determined according to the following formula:

Sphere formation efficiency (%) = (Number of spheres/Number of seeded cells) × 100

### 2.13. Flow Cytometry Assay

Flow cytometry was utilized to assess the levels of CD44^+^ and CD24^−^ cells in each experimental group. The evaluation was performed using specific antibodies conjugated with Allophycocyanin (APC) for CD44 (BioLegend, San Diego, CA, USA) and phycoerythrin (PE) for CD24 (BioLegend, San Diego, CA, USA). Isotype controls, including Rat IgG2a, κ-APC (BioLegend, San Diego, CA, USA), Mouse IgG2a, κ-PE (BioLegend, San Diego, CA, USA) were used (all antibodies diluted at a 1:100 ratio). Cells were incubated with antibodies at 4 °C for 45 min and analyzed using FACS Calibur™ (BD Biosciences, San Jose, CA, USA). The raw data were analyzed using Flowing Software version 2.5.0.

### 2.14. Western Blot Analysis

The expression levels of Bax, Bcl-2, and GAPDH as internal control were assessed using Western blot analysis using rabbit polyclonal anti-human Bax antibody (1:1500, Abcam, Cambridge, UK), rat monoclonal anti-human Bcl-2 antibody (1:1000, Abcam, Cambridge, UK), and rabbit polyclonal anti-human GAPDH (1:1000, Abcam, Cambridge, UK), as previously described [24].

### 2.15. Reverse Transcription–Quantitative (q-RT) PCR

Total RNA from cell lines and mammospheres in both control and treated groups was extracted using TRIzol reagent (Invitrogen, Paisley, UK). cDNA was generated from 1 μg of total RNA using a cDNA Synthesis Kit (Takara Bio, Inc., Shiga, Japan) according to the manufacturer’s instructions. The real-time PCR was conducted using 1X SYBR^®^ Green PCR Master Mix (Applied Biosystems, Foster City, CA, USA) and was performed in 48-well optical reaction plates. The expression of the EMT and stemness-associated genes *OCT4*, *c-MYC*, *KLF4*, *SOX2*, *NANOG*, *CDH1*, *ZO-1*, *CDH2*, *SNAIL1*, and *ZEB1* was assessed using a 7500 real-time PCR system (Applied Biosystems, CA, USA). The sequence of the used primers is provided in Table 1. The expression of the assessed genes was normalized relative to GAPDH, and data were analyzed using the 2-∆∆CT method [25].

### 2.16. Statistical Analyses

All experiments were performed at least in triplicate, the results were expressed as mean ± SD, and *p* ≤ 0.05 was considered statistically significant. Skewed and normal distributed metric variables were analyzed using Mann–Whitney U and one-way ANOVA tests, respectively, by using SPSS version 16 (SPSS Inc., Chicago, IL, USA).

## 3. Results

### 3.1. HPLC Results

In this research, aerial parts of *S. bachtiarica* were investigated. Accordingly, different amounts of polyphenolics were obtained based on the HPLC analysis (Table 2). In *S. bachtiarica*, rosmarinic acid was the predominant compound, followed by syringic acid, which was in line with previous reports on this species [15]. Among flavonoids, apigenin was revealed to exist in the highest amount in aerial parts of this species (Table 2).

### 3.2. S. bachtiarica Reduces the Viability of Adherent MDAMB-231 and U87-MG Cells and Induces Apoptosis

**Figure 1:**
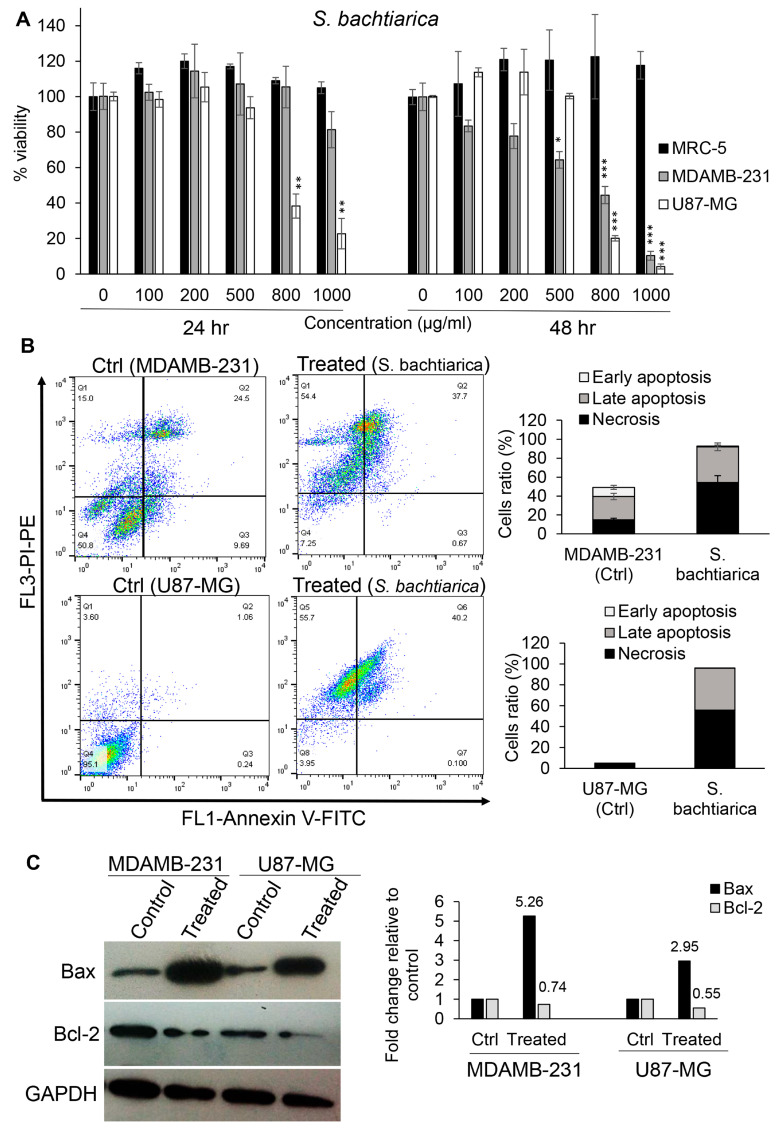
*S. bachtiarica* has anti-proliferation and apoptosis-inducing effects on MDAMB-231 and U87-MG cells. (**A**) MTT assay was used to assess viability of MDAMB-231 and U87-MG cells after treatment with different concentrations of *S. bachtiarica* for 24 and 48 h. (**B**) Representative scatterplots (left panel) and quantitative statistics of flow cytometry detection of *S. bachtiarica*-induced apoptosis (right panel) in MDAMB-231 and U87-MG cells at 48 h. (**C**) The protein expression of Bax and Bcl-2 was assessed in MDA-MB-231 and U87-MG cells after treatment with *S. bachtiarica* using Western blot analysis (left and right panel). GAPDH was used as internal control. Data are presented as the mean ± standard deviation of three different biological repeats. * *p* < 0.05, ** *p* < 0.01, *** *p* < 0.001.

To determine the cytotoxic effect of *S. bachtiarica* on MRC-5, MDAMB-231, and U87-MG cell lines, all cells were treated with different concentrations of *S. bachtiarica* for 24 and 48 h. The results showed that *S. bachtiarica* significantly reduced the cell viability of cancerous cell lines in a dose- and time-dependent manner (Figure 1A). Interestingly, no toxic effect was observed on normal fibroblastic MRC-5 cells in the presence of *S. bachtiarica* (Figure 1A). The IC_50_ dose of *S. bachtiarica* was determined to be 630 μg/mL and 680 μg/mL for MDA-MB-231 and U87-MG cells, respectively. Due to the ineffectiveness of the *S. bachtiarica* on normal fibroblast cells, the study was continued with an IC_50_ dose of *S. bachtiarica* on the MDAMB-231 and U87-MG cell lines. To investigate the effect of *S. bachtiarica* on apoptosis and determination of type of cell death, cells were treated with IC_50_ dose of *S. bachtiarica* for 48 h and then labeled with annexin V-FITC/PI and examined using flow cytometry. Treatment with *S. bachtiarica* caused the induction of apoptosis in MDAMB-231 cells, including early apoptosis (0.67% vs. 9.69% for control), late apoptosis (37.7% vs. 24.5% for control), and necrosis (54.4% vs. 15% for control) (Figure 1B, upper panel). Similar results with a higher intensity of apoptosis were observed in U87-MG cells as well, which showed early apoptosis (0.1% vs. 0.24% for control), late apoptosis (40% vs. 1.06% for control), and necrosis (55.7% vs. 3.6% for control) (Figure 1B, lower panel). Western blot analysis of Bax and Bcl-2 indicated that *S. bachtiarica* increases the expression of Bax in both MDAMB-231 and U87-MG cell lines, while the protein expression pf Bcl-2 was decreased in the presence of *S. bachtiarica* compared to the untreated group (Figure 1C, left and right panel).

### 3.3. S. bachtiarica Reduces the Growth Rate of MDAMB-231 and U87-MG Spheroids

**Figure 2:**
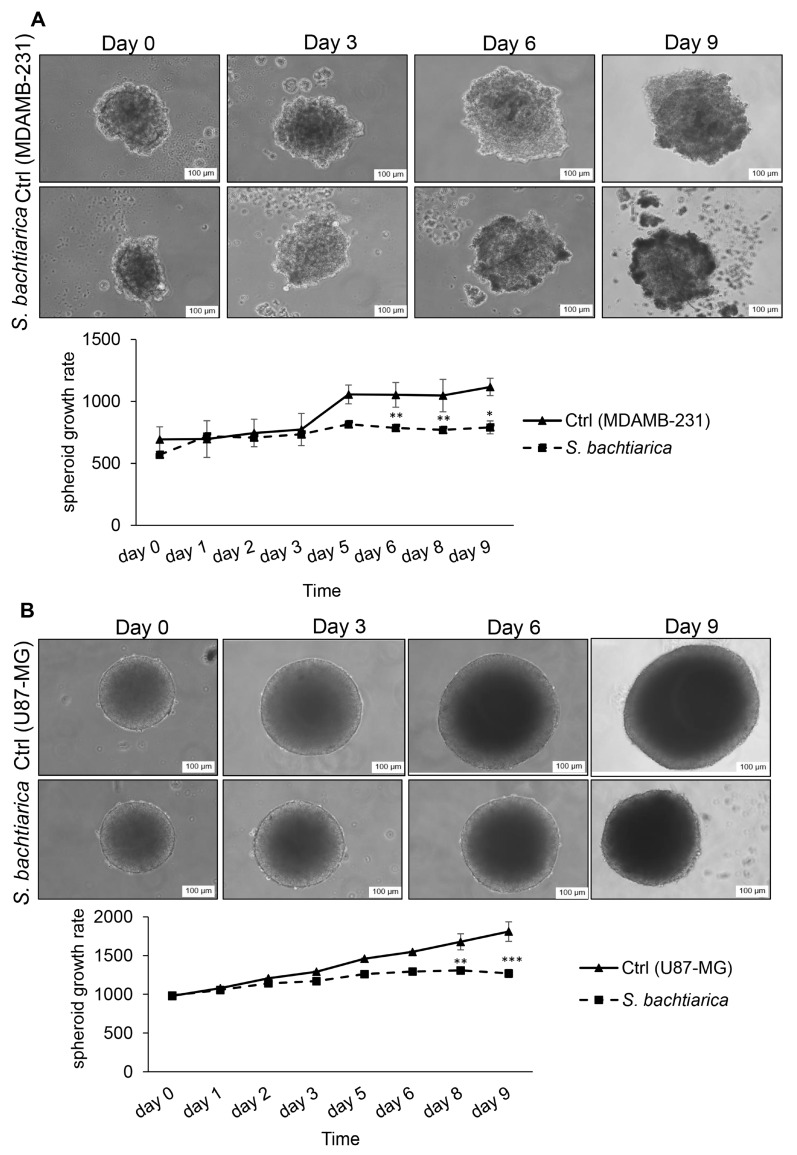
*S. bachtiarica* has growth inhibitory effects on MDAMB-231 and U87-MG spheroids. (**A**) Bright-field image of the MDAMB-231 spheroids (scale bar = 100 μm) after 9-day culture (upper panel), size growth rate of treated MDAMB-231 spheroids versus untreated spheroids during nine days (lower panel). (**B**) Size growth rate of treated U87-MG spheroids (scale bar = 100 μm) after 9-day culture (upper panel), quantitative statistics of growth rate of U87-MG spheroids versus untreated spheroids over nine days (lower panel). * *p* < 0.05, ** *p* < 0.01, *** *p* < 0.001.

To determine the growth inhibitory effect of *S. bachtiarica* on MDAMB-231 and U87-MG cells in a 3D model, cell spheroids were formed with the hanging drop technique and then spheroids were treated with IC_50_ dose of *S. bachtiarica* for nine days. The results showed that MDAMB-231 and U87-MG spheroids, exhibited significantly reduced growth rate during the nine days’ post-treatment (*p* < 0.05, *p* < 0.001, respectively, Figure 2A,B upper and lower panel, respectively). The inhibitory effect of *S. bachtiarica* on the growth rate of MDAMB-231 spheroids was more pronounced, leading to the disintegration of spheroids (Figure 2A, upper panel).

### 3.4. S. bachtiarica Decreases Clonogenic Growth of MDAMB-231 and U87-MG Cell Lines

**Figure 3:**
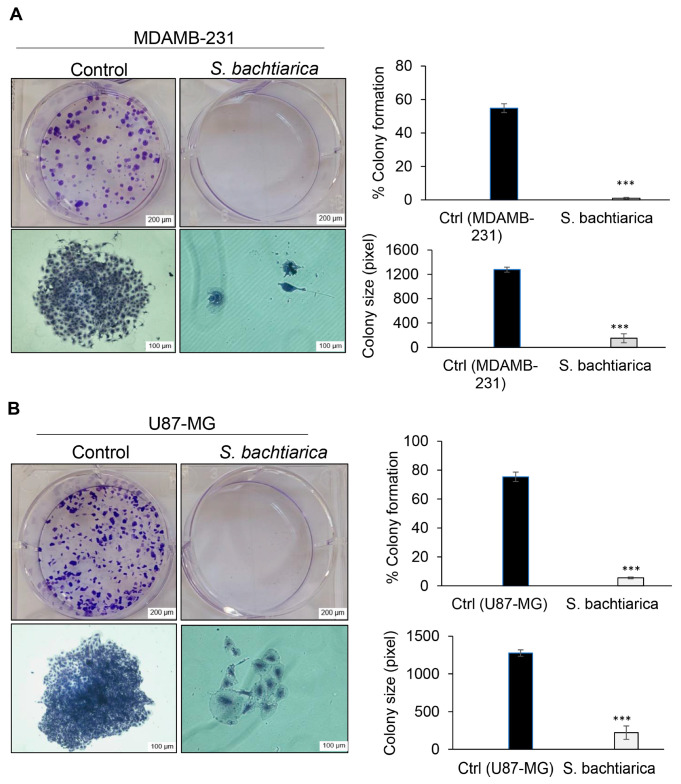
Decreased clonogenicity of *S. bachtiarica*-treated MDAMB-231 and U87-MG cell lines. (**A**) Representative images of colony formation and staining of a single colony with crystal violet in MDAMB-231 cell lines treated with IC_50_ dose of *S. bachtiarica* for 48 h (left panel, scale bar: 200 and 100 µm, respectively). The right panel represents a quantitative assessment of colony formation and size in the MDAMB-231 cells after 11 days. (**B**) Representative images of colony formation and staining of a single colony with crystal violet in U87-MG cell lines treated with IC_50_ dose of *S. bachtiarica* for 48 h (left panel, scale bar: 200 and 100 µm, respectively). The right panel represents a quantitative assessment of colony formation and size in the U87-MG cells after 11 days. (*** *p* < 0.001 relative to untreated cells).

In this study, in order to investigate the effect of *S. bachtiarica* on the clonogenic ability of cells, MDAMB-231 and U87-MG cells were treated with IC_50_ dose of *S. bachtiarica* for 48 h and then cells were cultured as single cells and the colony formation was followed for 11 days. We found a 55-fold and 13.6-fold reduction in the number of colonies in both MDAMB-231 and U87-MG cells, respectively (Figure 3A,B, right panel). Moreover, our data showed a smaller size of MDAMB-231 and U87-MG colonies compared to untreated cells (*p* < 0.001, Figure 3A,B left panel).

### 3.5. S. bachtiarica Inhibits MDAMB-231 and U87-MG Cell Motility and Invasion through DownRegulation of EMT Factors

**Figure 4:**
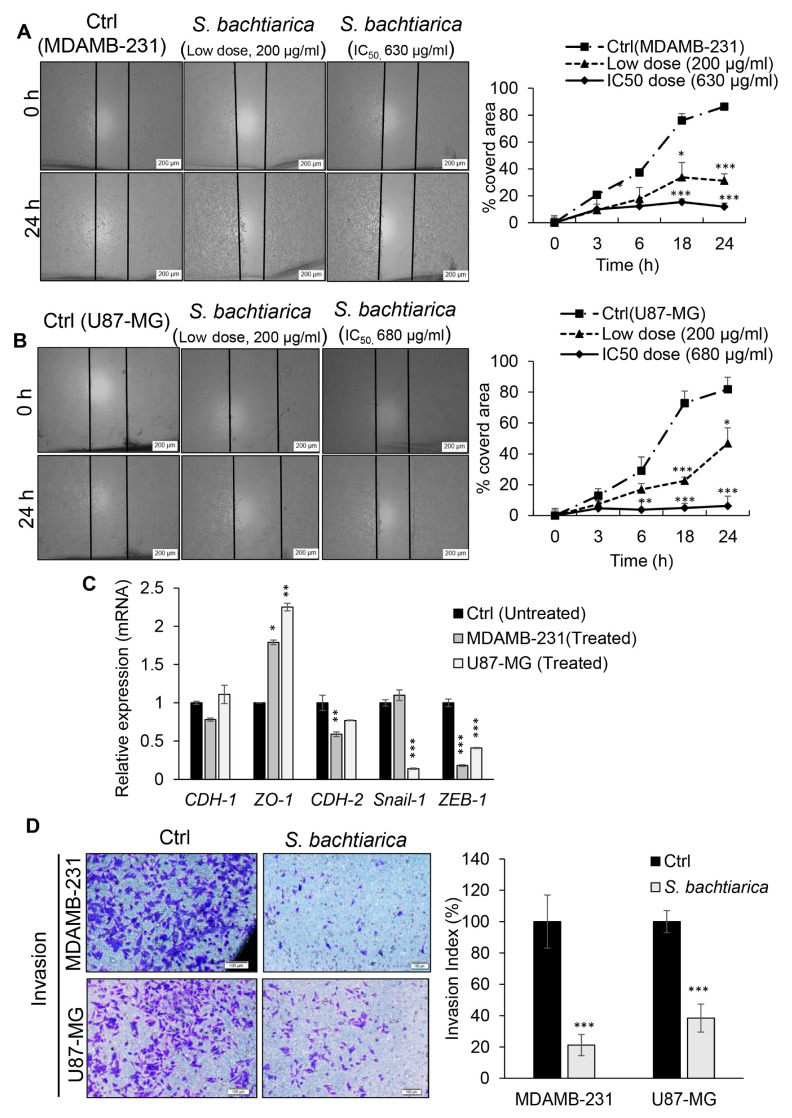
*S. bachtiarica* alters motility and invasiveness of MDAMB-231 and U87-MG cell lines by modulating EMT factors. (**A**) Representative images (left panel) and quantitative statistics (right panel) of MDAMB-231 cell-scratching assay with or without *S. bachtiarica* treatment (scale bar = 200 µm). (**B**) Representative images (left panel) and quantitative statistics (right panel) of U-87-MG cell-scratching assay with or without *S. bachtiarica* treatment (scale bar = 200 µm). (**C**) RT-qPCR analysis of *CDH-1*, *ZO-1*, *CDH-2*, *Snail-1* and *ZEB-1* in MDAMB-231 and U87-MG cell lines treated with or without IC_50_ dose of *S. bachtiarica* for 48 h. Results were normalized related to *GAPDH* gene expression and presented as mean ± SD. (**D**) Representative images (left panel) and quantitative statistics (right panel) of MDAMB-231 and U87-MG cell invasion assay with or without *S. bachtiarica* treatment (scale bar = 100 µm). (* *p* < 0.05; ** *p* < 0.01; *** *p* < 0.001 relative to untreated cells).

In accordance with the observed inhibitory effect of *S. bachtiarica* on the proliferation and growth rate of both cell types in the adherent and spheroid model and in order to gain a better understanding of the effect of *S. bachtiarica* on migration of cells, the scratch assay was performed with the assessment of the expression pattern of genes involve in EMT. We found that the MDAMB-231 cell line treated with *S. bachtiarica* at both low and IC_50_ doses showed a significant reduction in migration compared to the untreated group after 24 h (*p* < 0.001, Figure 4A, left and right panel). In U87-MG cells, the results were similar to breast cancer cells, and they showed a significant reduction in a gap closure 24 h after scratching (Figure 4B, left and right panel).

Due to the obvious effect of *S. bachtiarica* in inhibiting the migration of MDAMB-231 and U87-MG; therefore, we investigated the expression of factors involved in EMT in both cell lines treated with *S. bachtiarica*. We found a significantly decreased level of *CDH-2* by 1.7-fold in MDAMB-231 cells, but this decrease was not significant in U87-MG cells. Similarly, *ZEB-1* expression levels were decreased by 5.6-fold in MDAMB-231 and by 2.43-fold in U87-MG cells (Figure 4C, *p* < 0.001). However, the expression levels of *Snail-1* were decreased by 86% only in U87-MG cells (Figure 4C, *p* < 0.001), but it did not show any change in breast cancer cells. The expression profile of *CDH-1* was different in both cell lines, showing a slight increase in the expression of *CDH-1* in the U87-MG cells, which was not significant. Whereas, there was a decreased level of *CDH-1* in MDAMB-231 cells (Figure 4C). The expression of *ZO-1* in both cell lines showed a significant increase compared to the control group (Figure 4C). Surprisingly, cell invasion of the MDAMB-231 and U87-MG cell lines was decreased by 79% and 61.5%, respectively, compared to untreated cells (Figure 4D left and right panel, *p* < 0.001).

### 3.6. S. bachtiarica Inhibits Disaggregation of MDAMB-231 and U87-MG Spheroids

**Figure 5:**
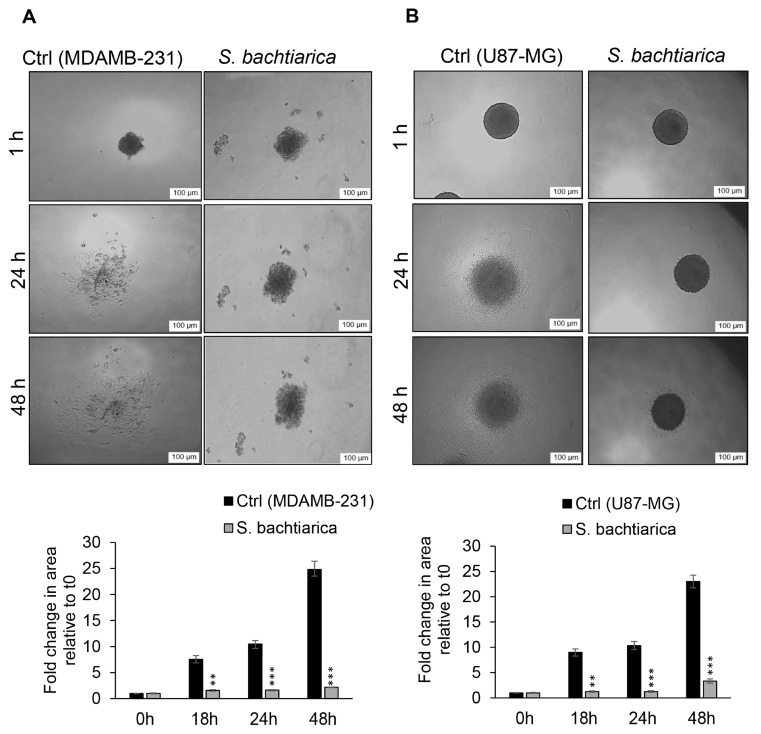
*S. bachtiarica* inhibits disaggregation of MDAMB-231 and U87-MG spheroids. (**A**) Representative images (upper panel) of MDAMB-231 spheroids on a matrigel-coated plate. Five to ten spheroids were transferred into matrigel-coated wells in the presence or absence of *S. bachtiarica*. Disaggregated spheroids formed a single-layer cell which derived from the peripheral layer of spheroids, lower panel; quantitative statistics of MDAMB-231 spheroid-disaggregating assay with or without *S. bachtiarica* treatment (scale bar = 100 µm). (**B**) Representative images (upper panel) of U87-MG spheroids on a matrigel-coated plate. Five to ten spheroids were transferred into matrigel-coated-wells in the presence or absence of *S. bachtiarica*. Disaggregated spheroids formed a single-layer cell which derived from the peripheral layer of spheroids, lower panel; quantitative statistics of U87-MG spheroid-disaggregating assay with or without *S. bachtiarica* treatment (scale bar = 100 µm). (** *p* < 0.01; *** *p* < 0.001 relative to untreated cells).

In disaggregation, spheroids become single-layer and cells migrate out of the spheroids. In this way, the contact of cells with each other decreases to adhere to and spread on the ECM components. Considering the inhibitory effect of *S. bachtiarica* on the migration of cells in the 2D model (Figure 4A,B), the effect of *S. bachtiarica* on the migration of cells in the 3D model of the spheroid was investigated. To this end, 6-day MDAMB-231 and U87-MG spheroids were seeded into matrigel-coated wells 18, 24, and 48 h in the presence or absence of IC_50_ dose of *S. bachtiarica*. In the absence of *S. bachtiarica*, we found that MDAMB-231 spheroids were rapidly disaggregated and formed a monolayer after 24 and/or 48 h while *S. bachtiarica* treated-MDAMB-231 spheroids remained compact after 24 and 48 h (Figure 5A upper and lower panel). Similarly, *S. bachtiarica* treated-U87-MG spheroids were not able to spread on matrigel, and a slight disaggregation occurred after 48 h (Figure 5B upper and lower panel), while untreated-U87-MG spheroids showed rapid disaggregation after 24 and 48 h. The extent of spheroid disaggregation on matrigel was quantitated by measuring the fold change in surface area of the spheroid over time relative to time t = 0 (Figure 5A,B lower panels). In both control spheroid group, the disaggregation on matrigel was significantly increased after 18 h, 24 h, or 48 h compared to time zero, but *S. bachtiarica*-treated-spheroids’ disaggregation remained unchanged over time (Figure 5A,B lower panels).

### 3.7. S. bachtiarica Reduces Stemness Potential in Breast Cancer Cell Line and Mammospheres

**Figure 6:**
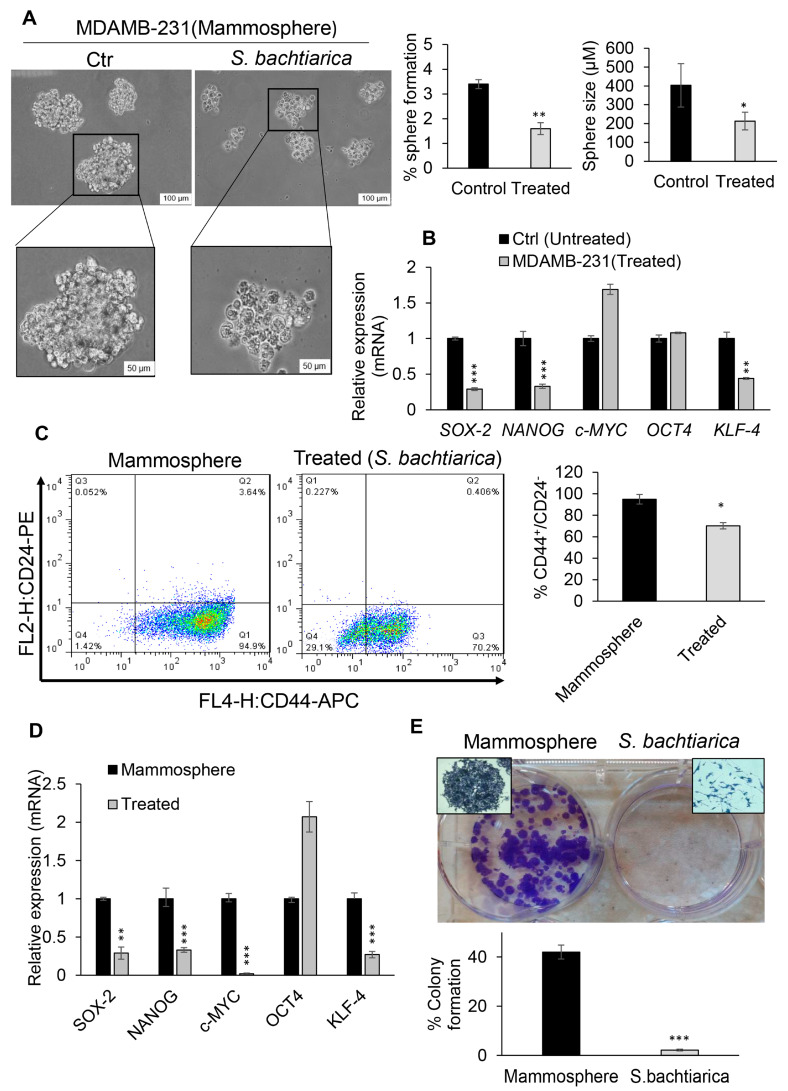
Decreased sphere formation and clonogenicity of mammospheres following *S. bachtiarica* treatment. (**A**) Representative images (left panel) of MDAMB-231-mammospheres and quantitative statistics, (right panel) of MDAMB-231-mammosphere formation and size with or without *S. bachtiarica* treatment. The results indicated the significant reduction in sphere formation ability and size of mammospheres after 48 h in both treated and untreated groups (scale bar: 100 and 50 μm). (**B**) RT-qPCR analysis of stemness genes *SOX-2*, *NANOG*, *c-MYC*, *OCT4*, and *KLF-4* in MDAMB-231 cell line treated with or without IC_50_ dose of *S. bachtiarica* for 48 h. Results were normalized related to GAPDH gene expression and presented as mean ± SD. (**C**) Evaluation of breast cancer stem cell markers in mammosphere cells treated with *S. bachtiarica*. Left panel; representative flow cytometry dot plots of CD24/CD44 stem cell markers in cells obtained from mammospheres, right panel; quantitative analysis of CD44/CD24 surface markers’ expression in treated and untreated mammospheres. (**D**) RT-qPCR analysis of stemness genes *SOX-2*, *NANOG*, *c-MYC*, *OCT4*, and *KLF-4* in mammospheres treated with or without IC_50_ dose of *S. bachtiarica* for 48 h. Results were normalized compared to *GAPDH* gene expression and presented as mean ± SD. (**E**) Representative images (upper panel) of colony formation in MDAMB-231-mammospheres treated with *S. bachtiarica* for 48 and the right panel represents a quantitative assessment of colony formation in the treated mammospheres after 11 days. (means ± SD; n = 3; * *p* < 0.05; ** *p* < 0.01; *** *p* < 0.001 relative to control).

An important characteristic of CSCs is a capacity to self-renew. To determine whether MDA-MB-231 and U87-MG cells exhibited this capacity in vitro and also in order to investigate the effect of *S. bachtiarica* on the sphere formation ability of these two cell lines, a sphere formation assay was performed. In the sphere formation assay, CSCs were the only cells which grew in serum-free medium on a non-adhesive surface [26,27]. Due to this, MDAMB-231 and U87-MG cell lines were treated with IC_50_ dose of *S. bachtiarica* for 48 h and then cells were cultured in serum-free medium on a non-adhesive surface. However, because of low efficiency of U87-MG cells in sphere formation, this cell line was removed from this section of the experiments. Here, we found that *S. bachtiarica* treatment led to a significant decrease by 2.12-fold and by 1.89-fold in sphere formation ability and size of MDAMB-231-mammospheres compared to untreated cells, respectively (*p* < 0.01 and *p* < 0.05, respectively, Figure 6A left and right panel). All these changes were associated with the downregulation of stemness genes *SOX-2* (*p* < 0.001), *NANOG* (*p* < 0.001) and *KLF-4* (*p* < 0.01) in treated MDAMB-231 cells in comparison to the untreated group (Figure 6B).

Subsequently, to gain a better understanding of the effect of *S. bachtiarica* on mammospheres, the 5-day MDAMB-231 mammospheres treated with *S. bachtiarica* for 48 h and the expression levels of surface markers associated with breast cancer stem cells, including CD44+/CD24− [28], the expression of stemness associated genes, including *OCT4*, *c-MYC*, *KLF-4*, and *SOX-2*, and the colonogenic capacity of mammospheres were assessed (Figure 6C–E). CD44 is a cell surface adhesion receptor that is highly expressed in many cancers and participates in cell adhesion migration and metastasis [29]. To determine the percentage of positive cells, the viable cell population was gated to use the forward-scattering and side-scattering results (Figure 6C, left panel). The percentage of CD44+/CD24- cells were significantly lower in the MDAMB-231-mammospheres treated with *S. bachtiarica* compared with the respective control (*p* < 0.05, Figure 6C, left and right panel),

Based on the above results, the expression of stemness-associated genes in mammospheres following treatment with *S. bachtiarica* was assessed. In treated mammospheres, the expression of all the stemness-associated genes was significantly downregulated, except for *OCT4* (*p* < 0.001, Figure 6D). Additionally, MDAMB-231-mammospheres treated with *S. bachtiarica* formed significantly fewer and much smaller colonies in comparison to untreated mammospheres (Figure 6E upper and lower panel).

## 4. Discussion

The presence of polyphenolic compounds in plant species provides new potential for different biological activities in plants. *S. bachtiarica* is one of the endemic species of Lamiaceae family, and the presence of high polyphenolic components like rosmarinic acid and syringic acid lead to an increase in the antioxidant and antiglycative capacities [15]. However, based on the previous research, phenolics and flavonoid compounds can also strengthen the anti-cancer capacity [30,31]. On this theme, in Rahimamlek et al.’s 2020 report, different concentrations of polyphenolic compounds were reported in Satureja species [15]. However, rosmarinic acid was higher (183.25 mg/100 gDW) in the present research (Table 2) in comparison with the previously reported study [15]. The previous reports also highlighted the anti-cancer role of rosmarinic acid [32,33], as well as syringic acid [34]. Studies have proven that the anticancer effects of rosemarinic acid can be through the induction of apoptosis, endoplasmic reticulum stress, G2/M cell cycle arrest, and the inhibition of cell migration and proliferation [32]. Syringic acid has also been proven to exert its anti-cancer effects by inhibiting proliferation, inducing apoptosis through increasing cellular ROS and DNA damage levels and the downregulation of major proliferative genes in vitro, as well as the reduction in tumor volume in vivo compared to the control [34]. As an exploratory study, the present study was designed to assess the anticancer potential of *S. bachtiarica* against the human glioblastoma and breast cancer. Until now, few studies have investigated the effect of *S. bachtiarica* on cancerous cells in a 3D model. Therefore, considering the importance of 3D culture models, especially in cancer research, the anti-cancer effect of *S. bachtiarica* was investigated in the 3D spheroid model in addition to the 2D culture model. Furthermore, the limited studies have focused on the abilities of plant compounds to target cancer stem cells. Therefore, the effect of *S. bachtiarica* on breast cancer stem cells (mammospheres) was also investigated. MTT assay and colony formation assay validated the anti-proliferative effect of *S. bachtiarica* on breast and glioblastoma cancer cells. The anti-proliferative effect of *S. bachtiarica* can be due to the induction of apoptosis in cells, as our results showed a high percentage of late apoptotic cells which was associated with an increase in Bax protein expression and a decrease in Bcl-2 protein level compared to the untreated group. The anti-proliferative and apoptosis-inducing effect of *S. bachtiarica* has been reported in very few studies, but we have found reports of similar action by extracts from other species of the Satureja genus. For instance, Asadipour et al. showed that different extracts of *S. bachtiarica* inhibited cell proliferation, which was mediated through the induction of a cell cycle arrest, apoptosis, and activation of caspase-3 in K562 and Jurkat leukemia cells [35]. Cakar et al. reported that the extracts of *S. subspicata* and *S. horvatii* induce apoptosis in human lymphocyte cell culture through the regulation of pro-apoptotic and anti-apoptotic genes *in vitro* and *in vivo* [36]. In addition, in another study, Esmaeili-Mahani et al. reported that *S. khuzestanica* extract induced apoptosis and prevented cell division in human breast cancer cells (MCF-7) [37]. Abdol et al. also reported similar results in which *S. sahandica* treatment led to the induction of apoptosis in hepatocellular carcinoma cell lines by regulation of apoptosis-related genes *BAX* and *BCL2* [38]. So far, no study has reported the effect of the Satureja genus on the growth of cells in a 3D culture model; our data reveal for the first time that *S. bachtiarica* reduces the growth of spheroids compared to the control group. Some studies have reported that compounds that can inhibit or reduce cell cycle progression can decrease the rate of invasion and metastasis of tumor cells [39,40]. In our study, cell scratch assay confirmed the ability of *S. bachtiarica* to inhibit the migration of glioblastoma and breast cancer cells. The reduction in cell motility was accompanied by the downregulation of mesenchymal markers involved in EMT, such as *CDH-2*, *Snail-1*, and *ZEB-1* (at mRNA level). In line with our results, Abdol et al. indicated that *S. sahandica* extraction significantly decreased the migration of cancer cells in a concentration-dependent manner [38]. Although there are few studies on the effect of the *Satureja* species on cancer cell migration and invasion, many researchers have studied the effect of polyphenolic components of this species on cell migration and invasion. For example, it has been shown that rosmarinic acid inhibits the migration and invasion of colorectal cancer cells through the upregulation of an epithelial marker, E-cadherin, and the downregulation of the mesenchymal markers, N-cadherin, snail, twist, vimentin, and slug, which is in line with the results of our study [41]. Syringic acid is one of the other polyphenolic components, the effects of which on the migration and invasion of cancer cells have been investigated. For example, Li et al. revealed that syringic acid prevents glioblastoma cells’ invasion and relocation through the downregulation of MMP-2 and -9 and upregulation of tissue inhibitors of metalloproteinases (TIMP1 and TIMP2) [42]. Another study showed that syringic acid considerably suppressed the cell invasion and cell migration potential of squamous cell carcinoma cells via the prevention of cell adhesion [43]. It is noteworthy to mention that no study has reported the effect of *Satureja* species on cell migration and invasion in a 3D model, and we have shown for the first time that *S. bachtiarica*-treated spheroids were not able to disaggregate on ECM components support their reduced invasiveness and motility potential.

Since CSCs perform pivotal roles in tumor initiation, progression, cell death resistance, therapy resistance, and tumor recurrence following treatment and remission, identifying and targeting them can effectively help in cancer treatment. Recently, many studies have focused on targeting cancer stem cells, However, there are very few studies that have investigated medicinal plant derivatives, and *Satureja* species and the effects of *S. bachtiarica* on cancer stem cells in particular were unknown. Therefore, the strength of this study is that it investigated the effect of *S. bachtiarica* on cancer stem cells. In the present study, mammospheres were used because they are rich in CSCs, [44,45,46] and the presence of a rare population of CSCs using CD44, CD24, and CD133 antibodies was confirmed in them. Spheroid culture conditions are more effective in enriching CSCs compared to cell surface markers [26,27]; therefore, mammospheres were used in this study. Our data showed that *S. bachtiarica* decreased the size and number of mammospheres and caused a downregulation of *SOX-2*, *KLF-4*, and *NANOG* as key transcription factors in the regulation of pluripotent ability. So far, there have been no studies on the effect of *Satureja* species and their derivatives on cancer stem cells, but several studies have investigated the effect of polyphenols on cancer stem cells [47,48].

One of the important findings in the present study is that treatment with *S. bachtiarica* decreased self-renewal potential, which was associated with a reduction in colony formation and mammosphere formation ability. Also, the proportion of CD44+/CD24− cells, which is an indicator of breast CSCs, were decreased in MDAMB-231-mammospheres. However, the expression of the stemness-associated genes *SOX-2*, *c-MYC*, *NANOG*, and *KLF-4* was downregulated. Similar results have been reported and shown that other polyphenols, including curcumin, silibinin, and quercetin and soy isoflavones such as genistein and phenylpropanoids, e.g., resveratrol glucosinolates sulforaphane, terpenoids, lycopene, piperine, and EGCG are capable of suppressing CSC progression, inhibiting their self-renewal potential and attenuating the acidic, hypoxic, and inflamed cancer micro-environment in various types of cancer [46,48,49,50,51,52,53,54,55,56]. The mechanism by which *S. bachtiarica* exhibits its effects are unknown.

Based on the reported findings and the current lack of understanding regarding the precise mechanism through which *S. bachtiarica* suppresses cancer cells and cancer stem cells, the assessment of the expression of proteins involved in the process of the colonization, migration, and invasion of the breast and glioblastoma cells after treatment with *S. bachtiarica* is recommended, as well as conducting further *in vivo* experiments to explore the mechanism of action of *S. bachtiarica* and its derivatives in suppressing cancer stem cells. Furthermore, it is important to explore their potential effects on tumorigenesis and metastasis. This research would provide valuable insights into the underlying mechanisms of *S. bachtiarica* and shed light on its therapeutic potential against cancer, particularly in relation to cancer stem cells.

## 5. Conclusions

In conclusion, taken together, our findings demonstrated the anticancer potential of *S. bachtiarica* against human glioblastoma and breast cancer, highlighting its anti-proliferative, apoptosis-inducing, and inhibitory effects on migration and invasion. The suppressive effect on cancer stem cells further emphasizes its significance in cancer treatment. Although the mechanism of action is not fully understood, the study suggests that *S. bachtiarica*’s bioactive compounds may act through various pathways. Overall, this research illuminates the potential of *S. bachtiarica* as a source of bioactive compounds for targeting cancer stem cells, warranting further investigation in vivo and in clinical settings.

## Figures and Tables

**Table 1:** Primer sequences used for reverse transcription–quantitative PCR.

Primer	Sequence
*NANOG*	F: 5′AGC TAC AAA CAG GTG AAG AC3′ R: 5′GGT GGT AGG AAG AGT AAA GG3′
*SOX-2*	F: 5′ATGCACCGCTACGACGTG 3′ R: 5′GCTGCGAGTAGGACATGCT 3′
*KLF-4*	F: 5′ATTACCAAGAGCTCATGCCA3′ R: 5′CCTTGAGATGGGAACTCTTTG3′
*OCT-4*	F: 5′TCT ATT TGG GAA GGT ATT CAG C3′ R: 5′ATT GTT GTC AGC TTC CTC CA3′
*Snail-1*	F: 5′ CCAGAGTTTACCTTCCAGCA 3′ R: 5′GATGAGCATTGGCAGCGA 3′
*c-MYC*	F:5′ACACATCAGCACAACTACG3′ R:5′CGCCTCTTGACATTCTCC3′
*CDH-1*	F: 5′GCTCTCCACTCTTACTTCCT3′ R: 5′GTTTGGTCTGATGCG3′
*CDH-2*	F: 5′GCCCAAGACAAAGAGACCC3′ R: 5′CTGCTGACTCCTTCACTGAC3′
*ZEB-1*	F: 5′CAGATGAAGCAGGATGTACAGTAA3′ R: 5′CTCTTCAGGTGCCTCAGGAA3′
*ZO-1*	F: 5′ACCAGTAAGTCGTCCTGATCC3′ R: 5′TCGGCCAAATCTTCTCACTCC3′

**Table 2:** Major phenolic and flavonoid compounds of *S. bachtiarica*. The values are expressed in mg/100 g of sample dry weight. The results are expressed as means ± SD.

Compounds	RT ^a^	*S. bachtiarica* (mg/100 gr DW)	*S. bachtiarica* (μg/1 mL Extract)
Gallic acid	5.08	1.32 ± 0.12	2.64
Caffeic acid	14.46	15.75 ± 0.32	31.5
Vanilic acid	16.23	n.d.	n.d.
Syringic acid	17.45	36.72 ± 2.13	73.44
*p*-Coumaric acid	26.8	12.86 ± 1.29	25.72
Rutin	28.41	13.41 ± 2.05	26.82
Ferulic acid	29.18	11.12 ± 1.11	22.24
Rosmarinic acid	39.05	183.28 ± 7.9	366.56
Luteolin	50.16	2.86 ± 0.27	5.72
Quercetin	50.64	5.23 ± 0.52	10.46
Apigenin	56.33	24.8 ± 3.15	49.6

^a^ Retention time of the compounds.

## Data Availability

The data presented in this study are available upon request from the corresponding author.
